# Degradation of Methyldopa by Banana

**DOI:** 10.3390/ph3030441

**Published:** 2010-03-02

**Authors:** Yoshihiro Uesawa, Kiminori Mohri

**Affiliations:** Clinical Pharmaceutics Laboratory, Meiji Pharmaceutical University, Japan

**Keywords:** banana, drug incompatibility, methyldopa

## Abstract

Methyldopa, an antihypertensive, is a very close analogue of DOPA. Drug interaction accompanied by degradation in a banana juice mixture was reported for DOPA. However, the effect of banana on methyldopa has not been reported. Therefore, we have investigated the impact of banana juice on methyldopa. The drug and supernatant of banana pulp were mixed, and the mixture was observed for changes in color, drug concentration, and ultraviolet-visible absorption spectra at 30 °C. The originally clear and colorless mixture started to acquire a yellow coloration after about 30 seconds after the mixing. The color tone increasingly deepened, then blistered solid particles that do not dissolve were observed after 3 hours. Concentration of methyldopa in the mixture decreased by 60% after 5 min, to 0.5% after 30 min of the mixing. From these findings, it was suggested that the drastic alterations were caused by banana polyphenol oxidase that plays a role in the biosynthesis of melanin pigment from levodopa in banana pulp. Because the degradation of methyldopa occurs extremely fast, it was suggested concomitant use of this anti-hypertensive and banana juice consumption should be avoided in clinical practice.

## 1. Introduction

**Figure 1:**
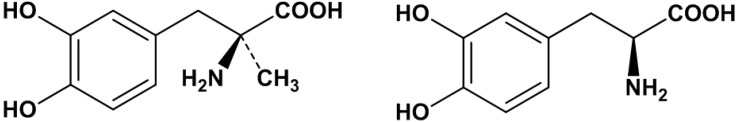
Chemical structures of methyldopa (left) and DOPA (right).

Methyldopa (MD) is an antihypertensive that controls the sympathetic nervous system via a central action [1]. This medication is typically administered to patients with heart failures, renal failures, and diabetes [2]. Furthermore, it is one of the few antihypertensives indicated in pregnancy-induced hypertension [2,3]. A structural feature of the drug is an amino acid skeleton with a catechol group as found in DOPA, an anti-Parkinsonism medication. In fact MD possesses a structure in which an α-hydrogen found in DOPA is replaced by a methyl group (Figure 1). It was reported by Garfinkel in 1972 that DOPA is degraded by mixing it with banana pulp [4]. He performed an investigation whereby powdered DOPA was mixed with banana at room temperature, and changes in the coloration of the mixture resulted – from a faint pink, followed by a bright brown, then a dark brown, then a gray, and, finally a liquorice-like black. Currently, there is substantial proof that indicates the alteration of color is caused by polyphenol oxidase (catechol oxidase; EC 1.10.3.1), an enzyme related with melanin biosynthesis, and found in banana pulp [5,6,7]. Furthermore, it was reported that the drug-incompatibility causes a reduction in bioavailability when the mixture is administered orally [8]. DOPA is a biological substrate in animals and plants that is involved in the melanin formation reaction [9,10]. Polyphenol oxidase rapidly oxidizes the catechol portion in the structure to DOPA quinone, then leucodopachrome, an irreversible intermediate in the melanin generation pathway, is formed non-enzymatically. Some of the following intermediates such as dopachrome (maximal absorption: 475 nm), 5,6-dihydroxyindole (296 nm), and melanochrome (540 nm) might be related with the color changes [9]. On the other hand, although MD has a catechol structure like DOPA, an interaction between MD and banana has not been reported. Therefore, we attempted to investigate a possible incompatibility of MD with banana.

## 2. Results and Discussion

### 2.1. Alteration in coloring

**Figure 2:**
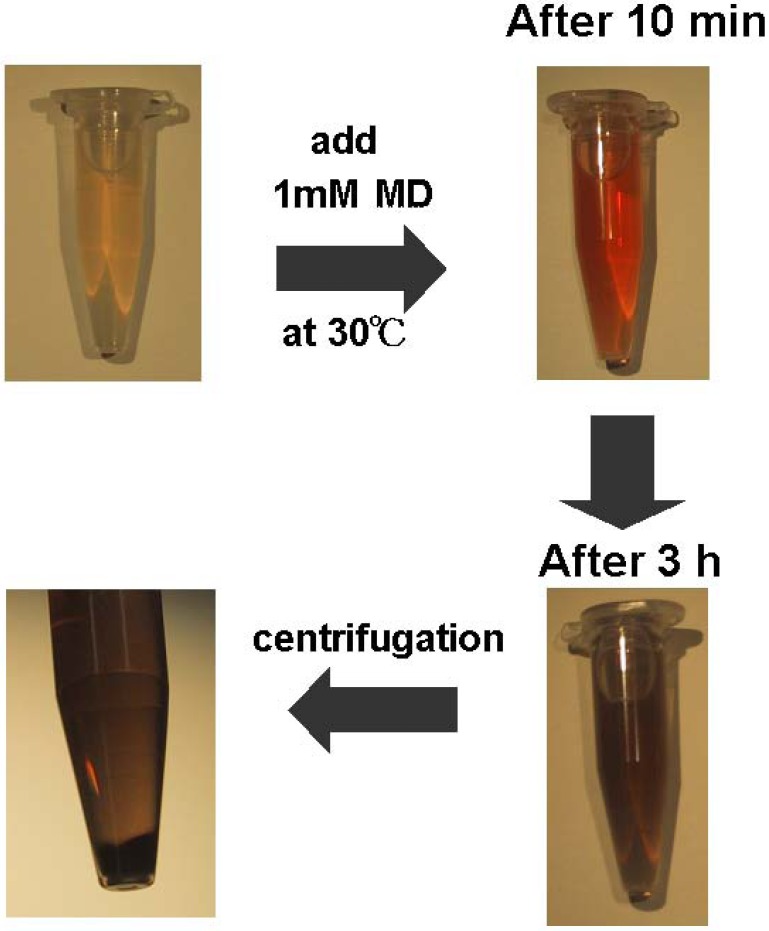
Alteration in coloring of banana supernatant with MD (1mM) during 3 h.

Alterations in the coloring of banana supernatant containing MD (1 mM) over 3 h are shown in Figure 2. Banana supernatant is transparent and almost colorless in the absence of MD. At 0.5 min after the addition of MD (final concentration of 1 mM), the coloring of the mixture started to change to yellow. After 10 min, the color was orange to red, then suddenly dusk brown after 30 min. After 3 h, an insoluble component was observed. A precipitate was also observed following centrifugation of the mixture at 3 h. These alterations of the coloring are very similar to the reported case of DOPA [4]. The coloring effect completely disappeared in an enzymatically-inactivated supernatant produced by autoclaving. In our previous experiments, large excess amounts of calcium chloride, magnesium sulfate, zinc sulfate, and copper sulfate were added to 1 mM MD to confirm the cause of coloring with a banana supernatant. The results showed no correlation between these metal ions and the coloring. These observations suggest that MD is a substrate of polyphenol oxidase in banana pulp as is DOPA. Generation of an insoluble component indicates that the polymerization reaction in melanin biosynthesis might be also progressed in the presence of MD.

### 2.2. Alteration in absorption spectrum

**Figure 3:**
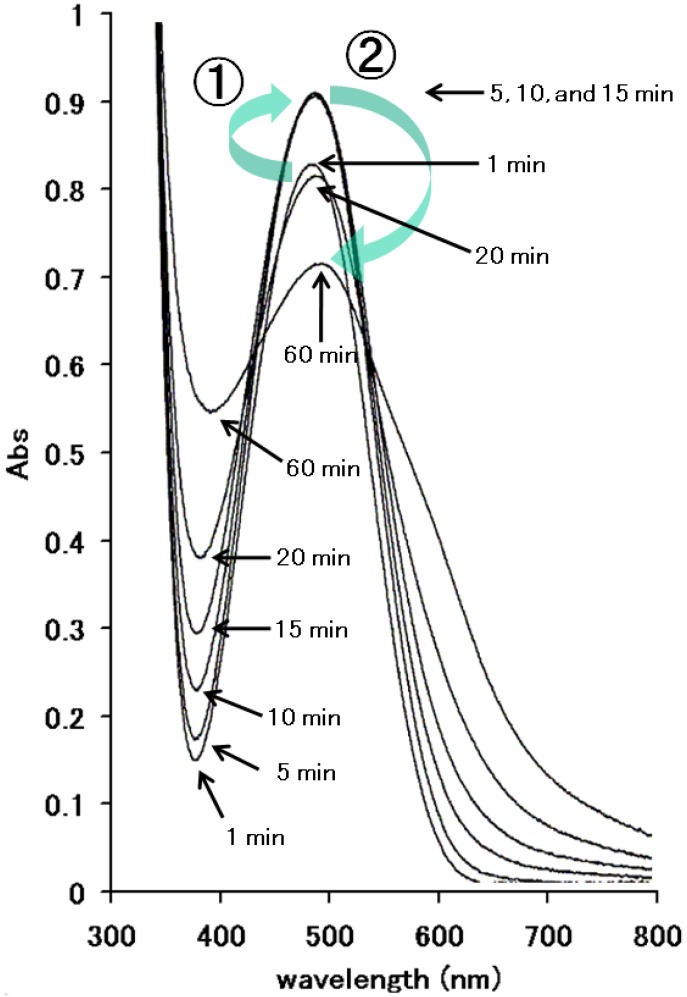
Alterations in the absorption spectrum of banana supernatant with MD at 30 °C at 1, 5, 10, 15, 30, and 60 min. Numbers 1 and 2 in the figure mean a change between 1min and 5 min and between 15 min and 60 min, respectively.

Alterations in the absorption spectrum of banana supernatant with MD at 30 °C during 60 min are shown in Figure 3. Absorbance at the maximal wavelength of the mixture (480 nm) increased until 15 min, and then decreased by 60 min. Such complicated changes of the spectrum indicate that the degradation reaction of MD with banana includes multiple products that contributed to the absorbance changes as is the case of the DOPA-banana reaction [4,9]. In our previous observations, spectra of MD in the absence of banana supernatant and the supernatant without MD were also scanned from 300 nm to 800 nm, and no peak was confirmed in both solutions. The peak at 480 nm in Figure 3 was from products of MD, but not MD itself, because scanning of MD from 300 nm to 800 nm doesn't show any obvious peaks. Furthermore, wavelengths of the peaks were slightly moved from 480 nm to 485 nm (60 min). These results indicate that the peak with the supernatant and MD in Figure 3 was dependent on the products following the reaction between MD and banana.

### 2.3. Alteration in MD concentration

**Figure 4:**
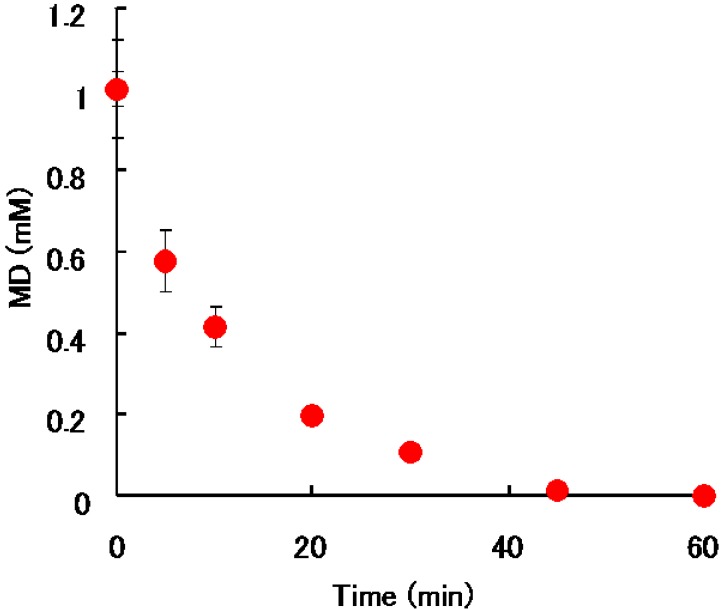
Alterations in concentration of MD in banana supernatant (BS) solution (1m M) at 30 °C at 0, 5, 10, 20, 30, 45, and 60 min after the addition of MD. Each point and vertical bar represents the mean ± SD of three experiments.

Alterations in concentration of MD in banana supernatant solution (1 mM) at 30 °C over a time span of 60 min are shown in Figure 4. Concentration of MD in the mixture decreased with time. The concentration after 5 min and 30 min of the addition of MD in the supernatant decreased to 60% and 0.5% of the initial concentration, respectively. It is considered that such a rapidly progressing degradation reaction would likely cause a significant interaction between MD and banana in clinical practice. 

## 3. Experimental Section

### 3.1. Materials

Methyldopa was obtained from LKT Laboratories, Inc. (St. Paul, MN, USA). Filipino banana was purchased from a local market in Japan. LC-MS grade acetonitrile was used (Wako Pure Chemical Industries Ltd., Osaka, Japan). All other chemicals were of reagent grade (Wako).

### 3.2. Preparation of banana supernatant

Banana pulp was minced and homogenized in distilled water at 50% or 10% (w/v) concentrations. The homogenate was centrifuged at 15,000 g for 10 min at 4 °C. Each supernatant was stored at −40 °C until use. Enzymatically inactivated supernatant from banana pulp was also produced by autoclave treatment (121 °C for 20 min) of the fresh banana juice supernatant.

### 3.3. Measurement of ultraviolet -visible absorption spectrum

At 30 °C, alteration of ultraviolet-visible absorption spectrum of 1 mM MD dissolved in 10% banana supernatant was measured by U-3210 Spectrophotometer (Hitachi High-Technologies Corporation, Tokyo, Japan). The absorbance of the mixture was scanned between 300 nm and 800 nm at 1, 5, 10, 15, 30, and 60 min after addition of MD into the supernatant. 

### 3.4. Measurement of MD concentration

MD (1 mM) was incubated in 50% supernatant of banana pulp at 30 °C. After 5, 10, 20, 30, 45, and 60 min of incubation, 100 times the volume of ice-cold acetonitrile was added to the reaction mixture. The sample was mixed vigorously for 20 s and centrifuged at 16,000 g for 5 min at 4 °C; then the supernatant (5 μL) was injected into LC/ESI/MS. ESI mass spectra were obtained using Shimadzu LCMS-2010EV LCMS system with an ESI probe (Shimadzu Co. Ltd., Kyoto, Japan) equipped with a Capcell Pak SCX UG80 column [2.0 mm (inside diameter) × 15 cm; particle size 5 μm (Shiseido Co. Ltd., Kyoto, Japan)]. The flow rate was set at 0.2 mL/min. The [M+H]^+^ ion at m/z 212.1 for MD was monitored for positive ions; the interface voltage was 4.5 kV, and the detector voltage was 1.5 kV. The heat block and CDL temperatures were 200 and 250 °C, respectively. Nitrogen was used as the nebulization gas at flow rates of 1.5 L/min. A mobile phase consisting of 3% acetonitrile in 1 mM ammonium formate was pumped through the column at a flow rate of 0.2 mL/min. Calibration curves (2 μM to 1 mM of MD) were constructed using regression analysis.

## 4. Conclusions

It was demonstrated that MD is degraded in the supernatant of banana pulp following mixing. The degradation reaction was accompanied by alterations of the coloring. Observation of the changes in the absorption spectrum revealed that the reaction produced multiple products with different absorbance wavelengths. These observations suggest that the degradation reaction of MD is caused by polyphenol oxidase, which is involved in melanin biosynthesis in plants, in banana as well as DOPA degradation. This drug-incompatibility might become a significant drug-food interaction because the reaction is rapidly progressive. As in the case of DOPA, MD might also have reduced bioavailability when administered concomitantly with banana juice [8]. The effects of intestinal absorption of MD when administered with banana should be clarified in *in vivo* experiments or clinical settings. MD should not be administered concomitantly with banana pulp to facilitate administration of the drug. 
